# Autonomic Nervous System Mediates the Hypotensive Effects of Aqueous and Residual Methanolic Extracts of *Syzygium polyanthum* (Wight) Walp. var. *polyanthum* Leaves in Anaesthetized Rats

**DOI:** 10.1155/2013/716532

**Published:** 2013-12-18

**Authors:** A. Ismail, M. Mohamed, S. A. Sulaiman, W. A. N. Wan Ahmad

**Affiliations:** ^1^School of Health Sciences, Health Campus, Universiti Sains Malaysia, 16150 Kubang Kerian, Kelantan, Malaysia; ^2^Department of Basic Medical Sciences, Kulliyyah of Dentistry, Kuantan Campus, International Islamic University Malaysia, Jalan Sultan Ahmad Shah, Bandar Indera Mahkota, 25200 Kuantan, Pahang, Malaysia; ^3^Department of Physiology, School of Medical Sciences, Health Campus, Universiti Sains Malaysia, 16150 Kubang Kerian, Kelantan, Malaysia; ^4^Department of Pharmacology, School of Medical Sciences, Health Campus, Universiti Sains Malaysia, 16150 Kubang Kerian, Kelantan, Malaysia

## Abstract

*Syzygium polyanthum *(Wight) Walp. var. *polyanthum* leaves are consumed as a traditional Malay treatment of hypertension. This study investigates hypotensive potential of aqueous (AESP) and residual methanolic (met-AESP) extracts of *S. polyanthum *leaves and possible involvement of autonomic receptors. AESP and met-AESP (20 to 100 mg/kg) were intravenously administered into anaesthetized Wistar-Kyoto (WKY) and spontaneously hypertensive (SHR) rats. Blood pressure and heart were monitored for 20 min. AESP and met-AESP induced significant dose-dependent hypotension, but only 100 mg/kg AESP caused mild bradycardia (*n* = 5). AESP-induced hypotension was more potent than that of met-AESP in WKY. AESP has a faster onset time than that of met-AESP in both WKY and SHR. However, met-AESP-induced hypotension was more sustained than that of AESP in SHR. Blockages of autonomic ganglion and **α**-adrenergic receptors using hexamethonium and phentolamine (*n* = 5 for each group) partially attenuated AESP-induced hypotension, suggesting involvement of **α**-adrenergic receptors. Blockages of autonomic ganglion, **β**-adrenergic, cholinergic receptors, and nitric oxide production using hexamethonium, propranolol, atropine, and N-**ω**-nitro-l arginine methyl ester (L-NAME) (*n* = 5 for each group) partially attenuated met-AESP-induced hypotension, suggesting involvement of **β**-adrenergic and cholinergic receptors via nitric oxide production.

## 1. Introduction

*Syzygium polyanthum* (Wight) walp. var.* polyanthum*, or known as “*serai kayu*” or “*salam*,” is consumed by Malays as a traditional remedy for hypertension. *S. polyanthum* leaves are well-known as traditional medication for various illnesses such as cataract, diarrhoea, gastritis, hypercholesterolemia, skin diseases, and diabetes mellitus [1]. Besides medicinal usages, the young shoots of *S. polyanthum* were commonly consumed as a fresh salad (*ulam*) whereas the mature leaves were regularly added as a flavour enhancer in Malays' cuisines.

With the popular use of *S. polyanthum *leaves, few studies on its biological properties were carried out. Among the findings, *S. polyanthum* leaves' extracts were proven to possess antibacterial activity against *Staphylococcus aureus* [2], antifungal activities against *Alternaria alternata *and *Colletotrichum capsici *[3], antinematodal activity against the pine wood nematode, *Bursaphelenchus xylophilus* [4], antitumor promoting activity [5], and antioxidant activity [6–9]. Besides, *S. polyanthum* leaves extract is also noncytotoxic to normal mammalian cell lines [9].

Despite its known biological effects, the phytochemical constituents in the crude extracts of *S. polyanthum *leaves were only preliminarily studied. The crude ethanolic extracts of the leaves and the fruits of *S. polyanthum* contain terpenoids, phenols, tannins, flavonoids, and alkaloids [8]. Steroids were found in the crude ethanolic extract of the leaves and the ripe fruits. Saponins were found in the unripe fruits, whereas carbohydrates were present in both the ripe and unripe fruits [8]. On the other hand, the chemical constituents of the essential oil from *S. polyanthum* leaves are extensively studied. Dalimartha (2000) in [10] reported eugenol as one of the compounds present in *S. polyanthum* leaves. Eugenol, a phenolic compound abundantly found in *Syzygium* family [11], has reputed ability as a vasorelaxant compound that causes vasodilation *in vitro* [12–14] and reduces blood pressure and heart rate of rats *in vivo* [15]. Other major phytochemical constituents of the essential oil from *S. polyanthum *leaves include cis-4-decenal, octanal, *α*-pinene, farnesol, *β*-ocimene, and nonanal [16]. While using hexane as solvent, the essential oil of *S. polyanthum* leaves contains cis-4-decenal, octanal, *α*-pinene, farnesol, nerolidol, and decanal at various percentages. Among the compounds, the presence of *α*-pinene which belongs to terpenoid family is notable since it was associated with hypotension in both the nonanaesthetized [17] and the urethane-anaesthetized rats [18]. Although these two compounds might correlate with the proclaimed traditional use of *S. polyanthum* leaves as an antihypertensive remedy, but these studied compounds were just sparingly soluble in water. Thus, the alleged claim on antihypertensive ability of the decoction of *S. polyanthum* leaves still requires verification.

Therefore, the aim of this study was to elucidate the effects of aqueous and residual methanolic extracts of *S. polyanthum* leaves on mean arterial (MAP), systolic (SBP), and diastolic (DBP) blood pressure and heart rate (HR) of anaesthetized male Wistar-Kyoto (WKY) and spontaneously hypertensive (SHR) rats. Instead of using noninvasive blood pressure measurement method that requires prior warming and restraining of the rats which significantly increased the baseline blood pressure of SHR due to stress [19], the effects of AESP and met-AESP were elucidated in this study in a more calm, resting anaesthesia condition. Indeed, the invasive measurement of blood pressure under anaesthesia was widely used in determining hypotensive or antihypertensive properties of plant extracts [20–23]. Besides, this study also aims to elucidate the possible involvement of autonomic nervous system (ANS) in mediating the hypotensive effects of the extracts.

## 2. Materials and Methods

### 2.1. Reagents

Dimethylsulfoxide (DMSO) and 95% methanol (v/v) were purchased from Merck, Malaysia. Hexamethonium bromide, isoproterenol hydrochloride, propranolol hydrochloride, acetylcholine chloride, atropine sulphate, phentolamine hydrochloride, methoxamine hydrochloride, and N-*ω*-nitro-l arginine methyl ester (L-NAME) were purchased from Sigma, USA. Sodium pentobarbital (Nembutal) was bought as an injectable solution (60 mg/mL, w/v) from Ceva-Sante Animale, France. Heparin (Heparinol-5000, Malaysia) was bought from Ain Medicare Sdn. Bhd, Malaysia.

### 2.2. Animals

Three- to five-month-old male normotensive Wistar-Kyoto (WKY) and spontaneously hypertensive rats (SHR) (280–350 g) were supplied by Animal Research and Service Centre, Health Campus, Universiti Sains Malaysia. The research methodology was approved by the Animal Ethics Committee, Universiti Sains Malaysia (USM/Animal Ethics Approval/2010/(59) (244)). These animals were kept in standard rat cages and allowed to acclimatize for 7 days in a standard environmental condition (25°C with 60–70% humidity) on a 12 hr light-dark cycle. Animals were given standard rat pellet (Chipsi Classic Heimtierbett, Germany) and tap water *ad libitum*.

### 2.3. Plant Material

*S. polyanthum* leaves were collected from the District of Bachok, Kelantan, Malaysia, from March to April 2010. The plant was identified by Dr. Richard Chung from Forest Research Institute Malaysia (FRIM). The herbal specimen (dried leaves) was deposited into FRIM herbarium (sample number: PID-171011-10).

### 2.4. Preparation of Extracts and Drugs

Four kilograms of *S. polyanthum* leaves was weighed using digital weighing balance (A&D HV-60KGL, Columbia), washed with distilled water, and dried in an incubator (Memmert GmbH + Co.KG, Germany) at a preset temperature of 50°C for 3 consecutive days. The dried leaves (1.74 kg) were ground into powder in a laboratory blender (WARING Commercial, USA) and the filtrate was sieved off by mechanical siever (No. 35). For extraction, 1.5 kg of the powdered sample was immersed in 15 L of distilled water and heated on hot plate (Erla EMS-HP-700, Illinois) at 80–90°C with continuous stirring for 30 min. The extract was then filtered through Whatman No. 41 filter paper (Whatman Schleicher and Schuell, Malaysia) and then lyophilized in freeze-dryer (ilShin, Korea). The lyophilized sample (147.15 g) was designated as the aqueous extract of *S. polyanthum* leaves (AESP). To extract the remaining less-polar compounds from the residue of the aqueous extraction, 1.4 kg of the residue was extracted using 14 L of 95% methanol (v/v) in Soxhlet apparatus (Favorit, Thailand) for 2 continuous cycles. The extract was then concentrated via rotary evaporator (Heidolph Rotavac, Germany) and dried in the incubator (Memmert GmbH + Co.KG, Germany) at a preset temperature of 50°C. The extract was then designated as the methanolic extract of *S. polyanthum* leaves (met-AESP) (26.04 g). AESP and met-AESP were finally kept in an air-tight bottle and stored in a refrigerator (National NR-B53FE, Malaysia) at 4°C until use. AESP and met-AESP were dissolved in 0.9% (w/v) normal saline but met-AESP was further added with 5% (v/v) DMSO. AESP and met-AESP were freshly prepared by dissolving the extracts in 1 mL of their respective vehicles. AESP and met-AESP solutions (final dose of 100 mg/kg) were further diluted with their respective vehicles to achieve the doses of 70, 40, 30, 20, and 10 mg/kg. AESP and met-AESP solutions were then homogenized using a homogenizer (Ultra-Turrax T25 Basic, Malaysia) at 24,000/min for 3 min. All drugs were dissolved in 0.9% normal saline except for phentolamine hydrochloride which was dissolved in 0.9% normal saline plus 5% dimethylsulfoxide (DMSO).

### 2.5. Effects of Extracts on MAP, SBP, DBP, and HR of Anaesthetized Rats

WKY (*n* = 5, each for AESP- and met-AESP-treated group) and SHR (*n* = 5, each for AESP- and met-AESP-treated group) were anaesthetized with 50mg/kg sodium pentobarbital via intraperitoneal injection according to previous studies [20, 22] before being placed on a thermally controlled heating table (37 ± 1°C). Anaesthetic condition was assessed by pinching the tail and the toe. The use of 50 mg/kg sodium pentobarbital was reported to not significantly affect the baselines of blood pressure and heart rate of SHR [19], but in WKY, only baseline of blood pressure was not significantly affected while the heart rate was increased [24] in comparison with conscious rats. Indeed, several hypotensive studies using similar anaesthetics scheme reported a nonsignificant difference for the magnitude of hypotensive and bradycardic effects between both anaesthetized and conscious WKY rats [20–22]. Upon tracheotomy, an endotracheal polyethylene tube was inserted into the incised trachea to prevent airway obstruction. The left jugular vein was cannulated for extracts' injection and the right common carotid artery was cannulated for MAP, SBP, DBP, and HR recordings using MP30 BIOPAC acquisition system (BIOPAC Systems Inc., USA) *via* pressure transducer (SS13L) and analyzed using BIOPAC Student Lab Pro v3.6.7. After 20 min of equilibration period, 0.2 mL of the respective vehicle was intravenously administered as a negative control followed by 0.2 mL of AESP or met-AESP (10, 20, 30, 40, 70, and 100 mg/kg). In between these doses, an additional 0.2 mL of heparinised (5 IU/mL) normal saline (0.9%, w/v) was flushed intravenously to prevent intravascular blood clotting. MAP, SBP, DBP, and HR responses were observed. From MAP, SBP, DBP, and HR recordings, the changes in MAP (ΔMAP), SBP (ΔSBP), (ΔDBP), and HR (ΔHR) from baseline values were calculated and expressed in percentage as described by Medeiros and colleagues [25] using the following formula.

(1) (MAP/SBP/DBP/HRbaseline−MAP/SBP/DBP/HRafter  extracts  administration)MAP/SBP/DBP/HRbaseline×100.

Time-course changes in MAP, SBP, DBP, and HR were also recorded and analyzed on minute-to-minute basis for 20 min. The recording time was set to be 20 min based on our preliminary study that showed that the longest recovery time was 17 min.

### 2.6. Effects of Autonomic Ganglion, *α*-, *β*-Adrenergic, and Cholinergic Receptors Blockage in MAP, SBP, DBP, and HR of Anaesthetized WKY

Pharmacological antagonistic studies [15, 23] using blockers were performed on 5 different sets of experiments. Specific ANS receptor antagonists such as hexamethonium bromide (10 mg/kg), phentolamine hydrochloride (2 mg/kg), propranolol hydrochloride (2 mg/kg), and atropine sulphate (2 mg/kg) were used to block the autonomic ganglion, *α*-adrenergic, *β*-adrenergic, and cholinergic receptors, respectively. To investigate the role of nitric oxide, N-*ω*-nitro-l arginine methyl ester, L-NAME (20 mg/kg), was used to block the endothelial nitric oxide synthase (eNOS) enzyme. Specific agonists for *α*-adrenergic, *β*-adrenergic, and cholinergic receptors such as methoxamine hydrochloride (50 *μ*g/kg), isoproterenol hydrochloride (1.2 *μ*g/kg), and acetylcholine chloride (5 *μ*g/kg), respectively, were used as positive controls to ensure sufficient blockages. In each of the experiments, 100 mg/kg AESP or met-AESP was introduced. The dose was chosen based on our preliminary experiments whereby only 100 mg/kg AESP caused significant bradycardia. To check for the involvement of autonomic ganglion and eNOS enzyme blockages, these boluses of injections were administered intravenously according to the following sequence; (i) test dose, (ii) blocker, and (iii) test dose. In order to check for the involvement of *α*-adrenergic, *β*-adrenergic, and cholinergic receptors, the sequence of treatments was as follows: (i) test dose, (ii) agonist, (iii) blocker, (iv) agonist, and (v) test dose.

### 2.7. Statistical Analyses

MAP, SBP, DBP and HR were expressed as mean ± standard deviation (SD). ED_50_ values for AESP- and met-AESP-induced reductions of MAP, SBP and DBP were derived from nonlinear regression equation and calculated using GraphPad PRISM version 5.01. Statistical analyses were performed using similar software. One-way ANOVA test was used to determine significant differences between multiple doses, whereas repeated measures 2-way ANOVA test was used to determine the differences between the responses over time. By comparing the averaged readings of MAP, SBP, DBP, and HR upon extracts' administration with the initial baseline value every minute for 20 min, recovery time was then allotted when the averaged readings of MAP, SBP, DBP, and HR were no longer significantly different as compared to the initial baseline value. A post hoc Bonferroni test was performed to compare the effects of the multiple doses over time. For antagonistic study, paired *t*-test was carried out. The value of *P* less than 0.05 was considered to be significant.

## 3. Results

### 3.1. Effects of AESP and Met-AESP on MAP, SBP, DBP, and HR

**Figure 1:**
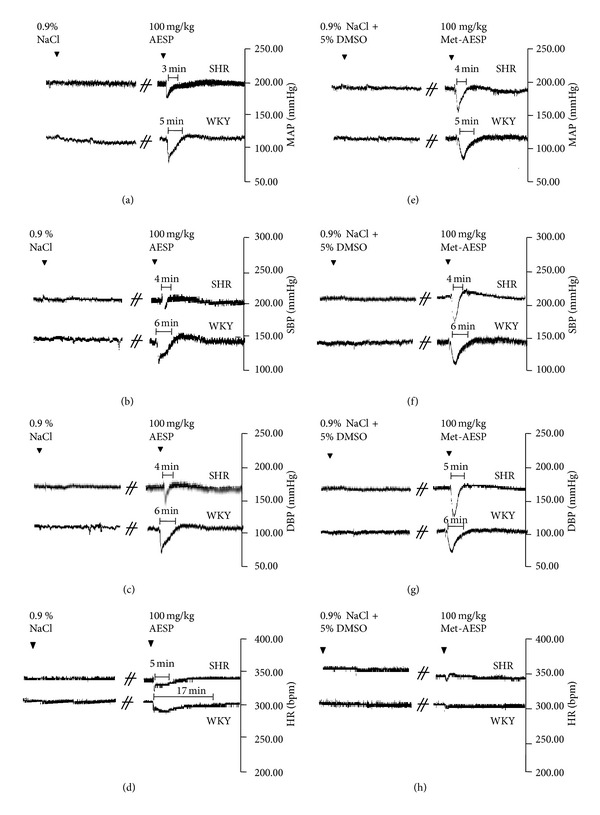
Tracings represent the effects of 0.9% normal saline as opposed to 100 mg/kg AESP on (a) MAP, (b) SBP, (c) DBP, and (d) HR and the effects of 0.9% normal saline plus 5% DMSO as opposed to 100 mg/kg met-AESP on (e) MAP, (f) SBP, (g) DBP, and (h) HR. MAP: recorded mean arterial blood pressure in millimetres of mercury, SBP: recorded systolic blood pressure in millimetres of mercury, DBP: recorded diastolic blood pressure in millimetres of mercury, HR: recorded heart rate in beats per minute, AESP: aqueous extract of *S. polyanthum* leaves, met-AESP: residual methanolic extract of *S. polyanthum *leaves, SHR: spontaneously hypertensive rats, WKY: Wistar-Kyoto rats, NaCl: sodium chloride, DMSO: dimethylsulfoxide.

Baselines of MAP, SBP, DBP, and HR for WKY before any treatment were 134.53 ± 10.72 mmHg, 150.20 ± 13.65 mmHg, 119.935 ± 9.27 mmHg, and 298.88 ± 42.17 beats/min (*n* = 10). Baselines of MAP, SBP, DBP, and HR in SHR were 194.25 ± 8.75, 218.14 ± 11.15 mmHg, 168.90 ± 11.89 mmHg, and 329.90 ± 30.32 bpm (*n* = 10). Intravenous administrations of 0.2 mL vehicles for AESP (0.9% normal saline) and met-AESP (0.9% normal saline + 5% DMSO) did not cause any significant changes to baseline MAP, SBP, DBP, and HR upon 20 min observation period. As compared to negative control, intravenous administrations of AESP bolus (0.2 mL, 100 mg/kg) caused significant changes to baseline MAP, SBP, DBP, and HR (see Figures 1(a), 1(b), 1(c), and 1(d)) while met-AESP bolus (0.2 mL, 100 mg/kg) caused significant changes to baseline MAP, SBP, and DBP (see Figures 1(e), 1(f), and 1(g)), but not to baseline HR (see Figure 1(h)) upon 20 min observation period.

**Figure 2:**
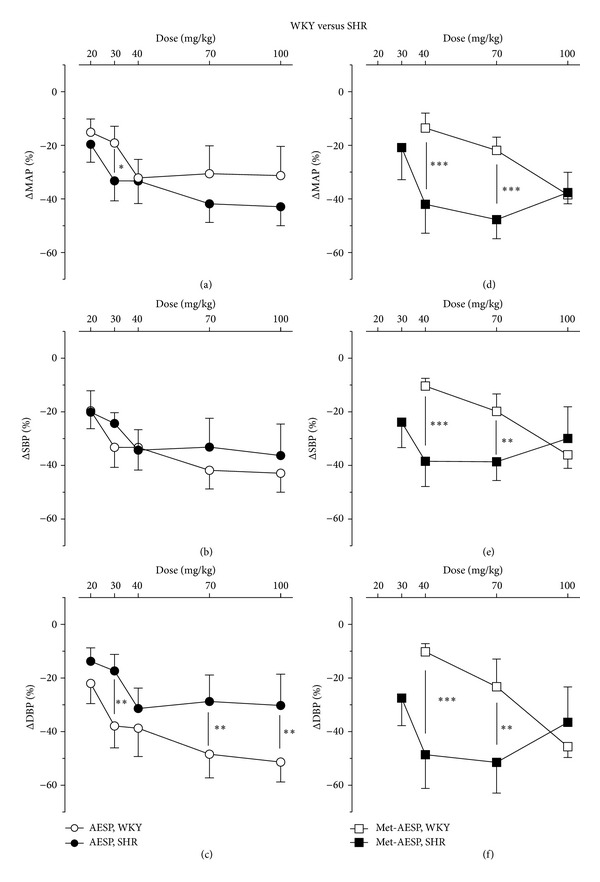
Effects of AESP on (a) MAP, (b) SBP, (c) DBP and effects of met-AESP on (d) MAP, (e) SBP, and (f) DBP of both WKY and SHR. ΔMAP/SBP/DBP: changes of MAP/SBP/DBP from baseline (expressed as mean percentage ± SD, *n* = 5 animals per group), AESP: aqueous extract of *S. polyanthum* leaves, met-AESP: residual methanolic extract of *S. polyanthum* leaves,WKY: Wistar-Kyoto rats, SHR: spontaneously hypertensive rats. *P < 0.05, **P < 0.01, and ***P < 0.001.

AESP from 20 to 100 mg/kg induced significant dose-dependent reductions of MAP (*P* < 0.001; see Figure 2(a)), SBP (*P* < 0.01; see Figure 2(b)), and DBP (*P* < 0.05; see Figure 2(c)) with only 100 mg/kg AESP causing a significant reduction of HR (*P* < 0.001; see Figure 4) in both WKY and SHR. AESP-induced reduction of MAP in WKY was significantly higher (*P* < 0.05) than in SHR at 30 mg/kg (see Figure 2(a)). AESP-induced reduction of SBP in WKY was comparable to reduction in SHR (see Figure 2(b)). AESP-induced reductions of DBP in WKY were significantly higher than in SHR at 30 (*P* < 0.01), 70 (*P* < 0.01), and 100 mg/kg (*P* < 0.01) (see Figure 2(c)).

Met-AESP from 40 to 100 mg/kg induced significant (*P* < 0.05) dose-dependent reductions of MAP (*P* < 0.05, see Figure 2(c)), SBP (*P* < 0.05; see Figure 2(d)) and DBP (*P* < 0.05; see Figure 2(e)) in WKY; whereas, in SHR, 30 mg/kg to 100 mg/kg met-AESP induced significant reductions of MAP (*P* < 0.001; see Figure 2(d)), SBP (*P* < 0.001; see Figure 2(e)) and DBP (*P* < 0.001, see Figure 2(f)). Met-AESP-induced reductions of MAP, SBP and DBP in SHR were significantly higher than in WKY at 40 (*P* < 0.001, *P* < 0.001, and *P* < 0.001, resp.) and at 70 mg/kg (*P* < 0.001, *P* < 0.01, and *P* < 0.01, resp.) (see Figures 2(d), 2(e), and 2(f)).

**Figure 3:**
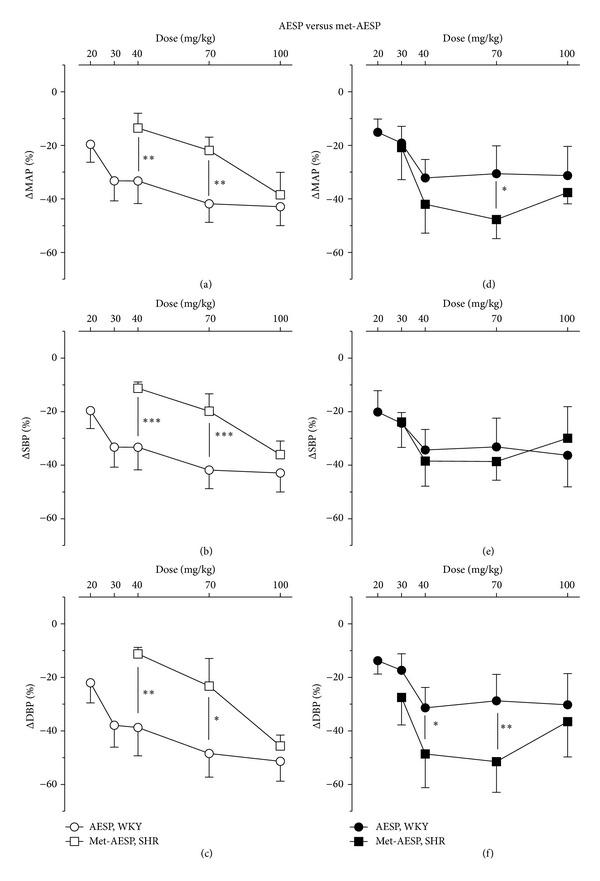
Effects of AESP and met-AESP on (a) MAP, (b) SBP, and (c) DBP of WKY and on (d) MAP, (e) SBP, (f) DBP of SHR. ΔMAP/SBP/DBP: changes of MAP/SBP/DBP from baseline (expressed as mean percentage ± SD, *n* = 5 animals per group), AESP: aqueous extract of *S. polyanthum* leaves, met-AESP: residual methanolic extract of *S. polyanthum* leaves, WKY: Wistar-Kyoto rats, SHR: spontaneously hypertensive rats. *P < 0.05, **P < 0.01, and ***P < 0.001.

AESP-induced reductions of MAP, SBP, and DBP were significantly higher than those of met-AESP-induced hypotension in WKY at 40 (*P* < 0.01, *P* < 0.001, and *P* < 0.01, resp.) and at 70 mg/kg (*P* < 0.01, *P* < 0.001, and *P* < 0.05, resp.) (see Figures 3(a), 3(b), and 3(c)). Met-AESP-induced reduction of MAP was significantly higher than AESP-induced reduction of MAP in SHR at 70 mg/kg (*P* < 0.05) (see Figure 3(d)). Met-AESP-induced reduction of SBP in SHR was comparable to AESP-induced reduction of SBP in SHR (see Figure 3(e)). Met-AESP-induced reductions of DBP were significantly higher than AESP-induced reduction of DBP in SHR at 40 mg/kg (*P* < 0.05) and 70 mg/kg (*P* < 0.01) (see Figure 3(f)).

ED_50_ value for AESP-induced reductions of MAP, SBP, and DBP was lower in WKY (25.28 ± 2.33, 21.80 ± 2.34, and 26.67 ± 2.34, resp.) than in SHR (32.28 ± 2.53, 26.32 ± 2.52, and 32.97 ± 2.58, resp.) (see Table 1). ED_50_ values for met-AESP-induced reduction of MAP, SBP and DBP were lower in SHR (33.05 ± 2.34, 32.08 ± 2.36, and 32.25 ± 2.34, resp.) than in WKY (54.58 ± 2.41, 63.83 ± 2.37, and 72.14 ± 2.43, resp.). Overall, the ED_50_ values for AESP-induced reductions of MAP, SBP and DBP were lower than those for met-AESP in WKY (see Table 1). The ED_50_ values for AESP-induced reductions of MAP and SBP were lower than ED_50_ values for met-AESP in SHR but not for DBP reduction (see Table 1).

**Figure 4:**
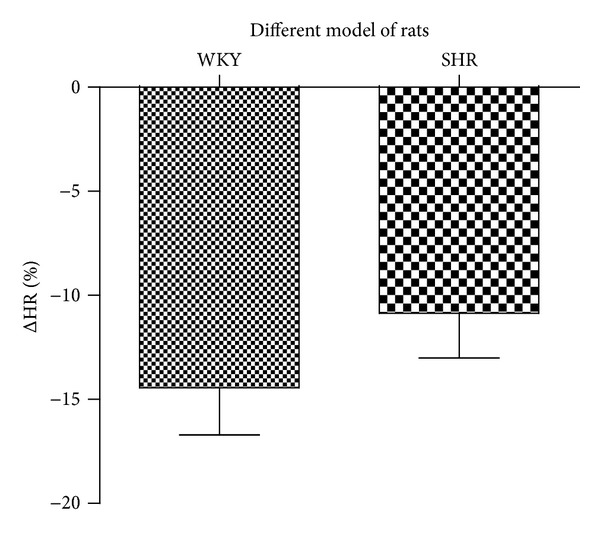
Effects of intravenous administrations of 100 mg/kg AESP on the heart rate of WKY and SHR. ΔHR: changes of heart rate from baseline (expressed as mean percentage ± SD), AESP: aqueous extract of *S. polyanthum* leaves, WKY: Wistar-Kyoto rats, SHR: spontaneously hypertensive rats.

Only 100 mg/kg AESP caused reduction of HR in both WKY and SHR. However, AESP-induced HR reductions in both WKY and SHR were not significantly different (see Figure 4).

### 3.2. Time-Course Changes in MAP, SBP, DBP, and HR by AESP and Met-AESP

In terms of the onset time of action, the maximal AESP-induced hypotension at all doses was achieved within 0.5 min after injection and for met-AESP was within 1.5 min.

Generally, AESP-induced reductions of MAP, SBP, and DBP recovered within 2–6 min in WKY and within 1–4 min in SHR. The highest dose of AESP (100 mg/kg) caused reduction of HR that recovered within 17 min in WKY and 5 min in SHR (see Table 2). Met-AESP-induced reductions of MAP, SBP and DBP recovered within 4–6 min in WKY and within 3–5 min in SHR (see Table 2). Generally, AESP recovered faster in SHR than in WKY. AESP also recovered faster than met-AESP in SHR.

### 3.3. Effects of Autonomic Ganglion Receptors Blockage on Changes in MAP, SBP, DBP and HR of Anaesthetized WKY

**Figure 5:**
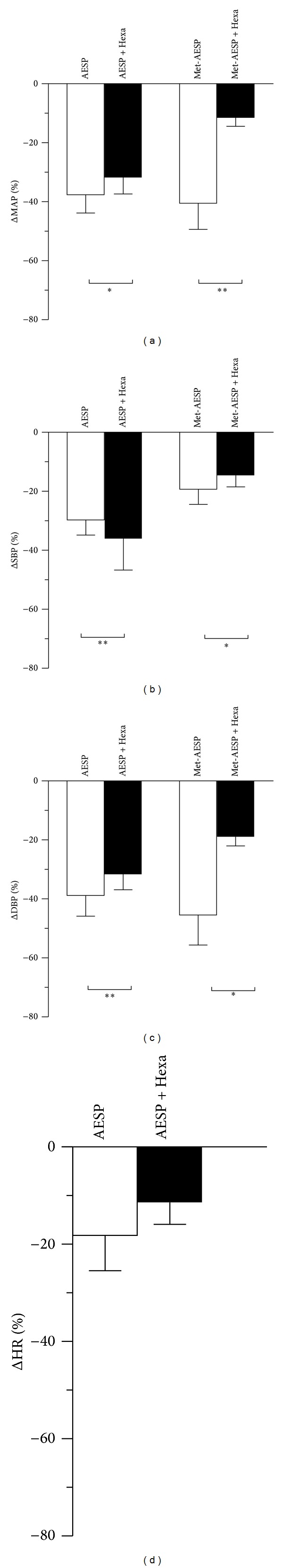
Effects of autonomic ganglion receptors' blockages on changes in (a) MAP, (b) SBP, (c) DBP, and (d) heart rate by various treatments in WKY rats (*n* = 5). Hexa: 10 mg/kg hexamethonium bromide, ΔMAP/SBP/DBP/HR: changes of MAP/SBP/DBP/HR from baseline (expressed as mean percentage ± SD), AESP: aqueous extract of *S. polyanthum* leaves, met-AESP: residual methanolic extract of *S. polyanthum *leaves, WKY: Wistar-Kyoto rats, SHR: spontaneously hypertensive rats. *P < 0.05 and **P < 0.01.

Blockage of autonomic ganglion with 10 mg/kg hexamethonium bromide caused significant attenuation in AESP-induced reductions of MAP, SBP, and DBP by 15.11 ± 10.01% (*P* < 0.05), 34.89 ± 14.77% (*P* < 0.01) and 18.21 ± 10.10% (*P* < 0.01), respectively (see Figures 5(a), 5(b), and 5(c)). Similar blockages have also attenuated the met-AESP-induced reductions of MAP, SBP, and DBP by 70.51 ± 10.84% (*P* < 0.01), 51.79 ± 19.43% (*P* < 0.05) and 47.20 ± 23.90% (*P* < 0.05), respectively (see Figures 5(a), 5(b), and 5(c)). The bradycardic effect by 100 mg/kg AESP was not significantly changed (see Figure 5(d)).

### 3.4. Effects of *α*-Adrenergic Receptors Blockage on Changes in MAP, SBP, DBP, and HR of Anaesthetized WKY

**Figure 6:**
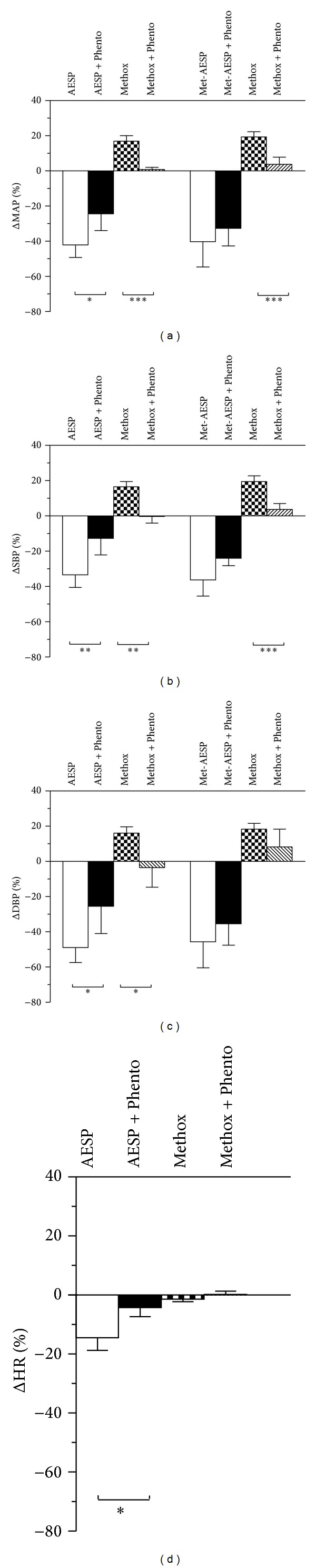
Effects of *α*-adrenergic receptors' blockage on changes in (a) MAP, (b) SBP, (c) DBP, and (d) HR by various treatments in WKY rats (*n* = 5). Phento: 2 mg/kg phentolamine hydrochloride, Methox: 50 *μ*g/kg methoxamine hydrochloride, ΔMAP/SBP/DBP: changes of MAP/SBP/DBP/HR from baseline (expressed as mean percentage ± SD), AESP: aqueous extract of *S. polyanthum* leaves, met-AESP: residual methanolic extract of *S. polyanthum *leaves, WKY: Wistar-Kyoto rats, SHR: spontaneously hypertensive rats. *P < 0.05, **P < 0.01, and ***P < 0.001.

Phentolamine hydrochloride (2 mg/kg) that significantly blocked the increments of MAP, SBP, and DBP induced by the positive control drug, methoxamine hydrochloride (50 *μ*g/kg), by 95.13 ± 7.17% (*P* < 0.001), 91.18 ± 14.76% (*P* < 0.001) and 87.23 ± 23.77 (*P* < 0.001) caused significant attenuations in AESP-induced reductions of MAP, SBP, DBP, and HR by 40.86 ± 24.34% (*P* < 0.05), 67.09 ± 23.69% (*P* < 0.01), 52.98 ± 31.62% (*P* < 0.05), and 70.35 ± 19.54% (*P* < 0.05), respectively (see Figures 6(a), 6(b), 6(c), and 6(d)). In another set of experiments, phentolamine hydrochloride (2 mg/kg) that significantly abolished the increment of MAP, SBP and DBP induced by the positive control drug, methoxamine hydrochloride (50 *μ*g/kg), by 80.80 ± 18.61% (*P* < 0.001), 68.16 ± 27.43% (*P* < 0.001), and 58.44 ± 38.61% (*P* < 0.001) did not significantly affect met-AESP-induced reductions of MAP, SBP, and DBP (see Figures 6(a), 6(b), and 6(c)).

### 3.5. Effects of *β*-Adrenergic Receptors Blockage on Changes in MAP, SBP, DBP, and HR of Anaesthetized WKY

**Figure 7:**
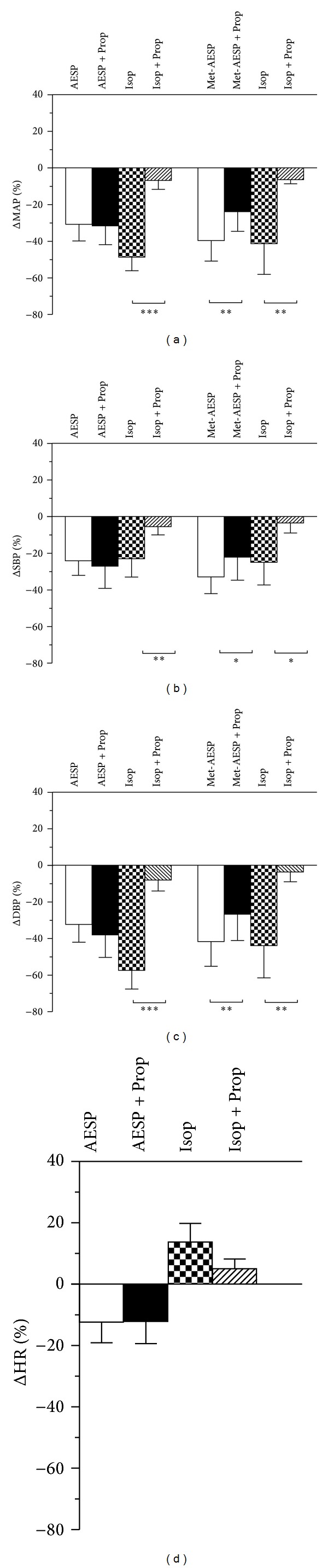
Effects of *β*-adrenergic receptors' blockage on changes in (a) MAP, (b) SBP, (c) DBP, and (d) HR by various treatments in WKY rats (*n* = 5). Prop: 2 mg/kg propranolol hydrochloride, Isop: 1.2 *μ*g/kg isoproterenol hydrochloride, ΔMAP/SBP/DBP/HR: changes of MAP/SBP/DBP/HR from baseline (expressed as mean percentage ± SD), AESP: aqueous extract of *S. polyanthum* leaves, met-AESP: residual methanolic extract of *S. polyanthum *leaves, WKY: Wistar-Kyoto rats, SHR: spontaneously hypertensive rats. *P < 0.05, **P < 0.01, and ***P < 0.001.

Propranolol hydrochloride (2 mg/kg) that significantly blocked the induced-reductions of MAP, SBP, and DBP by the positive control drug, isoproterenol hydrochloride (1.2 *μ*g/kg), by 85.52 ± 7.17% (*P* < 0.001), 76.17 ± 21.09% (*P* < 0.01), and 86.44 ± 9.90% (*P* < 0.001) did not significantly affect AESP-induced reductions of MAP, SBP, DBP, and HR (see Figures 7(a), 7(b), and 7(c)). In another set of experiments, propranolol hydrochloride (2 mg/kg) that significantly blocked the induced-reductions of MAP, SBP, and DBP by the positive control drug, isoproterenol hydrochloride (1.2 *μ*g/kg), by 80.30 ± 14.03% (*P* < 0.01), 73.63 ± 21.17% (*P* < 0.05), and 86.12 ± 10.18% (*P* < 0.01) also caused significant attenuations on met-AESP-induced reductions of MAP, SBP, and DBP by 39.27 ± 18.72% (*P* < 0.01), 33.87 ± 24.94% (*P* < 0.05), and 37.86 16.98% (*P* < 0.01), respectively (see Figures 7(a), 7(b), and 7(c)).

### 3.6. Effects of Cholinergic Receptors Blockage on Changes in MAP, SBP, DBP, and HR of Anaesthetized WKY

**Figure 8:**
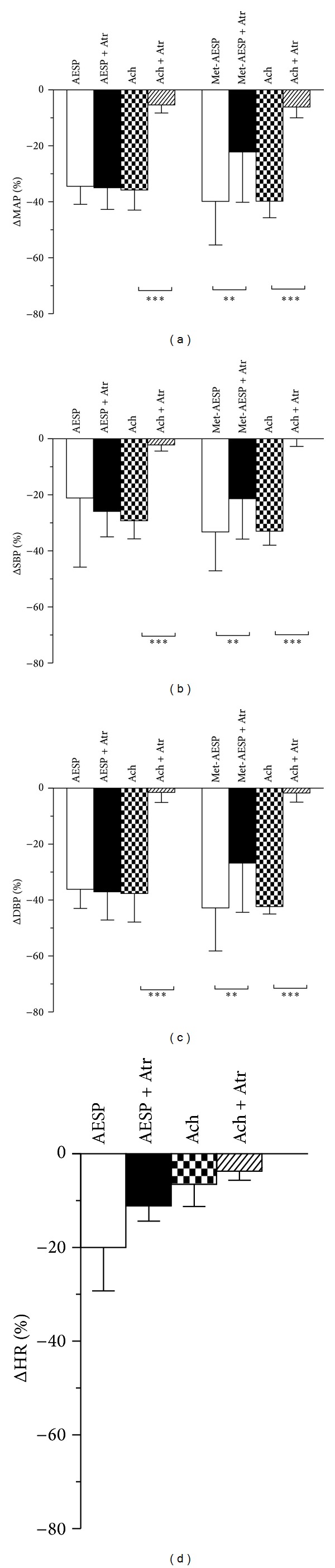
Effects of cholinergic receptors blockage on changes in (a) MAP, (b) SBP, (c) DBP, and (d) HR by various treatments in WKY rats (*n* = 5). Atr: 2 mg/kg atropine sulphate, Ach: 5 *μ*g/kg acetylcholine chloride. ΔMAP/SBP/DBP/HR: changes of MAP/SBP/DBP/HR from baseline (expressed as mean percentage ± SD), AESP: aqueous extract of *S. polyanthum* leaves, met-AESP: residual methanolic extract of *S. polyanthum *leaves, WKY: Wistar-Kyoto rats, SHR: spontaneously hypertensive rats. **P < 0.01 and ***P < 0.001.

Atropine sulphate (2 mg/kg) that significantly blocked the reductions of MAP, SBP, and DBP induced by the positive control drug, acetylcholine chloride (5 *μ*g/kg), by 85.77 ± 6.19% (*P* < 0.001), 96.07 ± 3.85% (*P* < 0.001), and 94.35 ± 4.76% (*P* < 0.001) did not significantly affect AESP-induced reductions of MAP, SBP, DBP and HR (see Figures 8(a), 8(b), 8(c), and 8(d)). In another set of experiments, atropine sulphate (2 mg/kg) that significantly blocked the reductions of MAP, SBP, and DBP induced by the positive control drug, acetylcholine chloride (5 *μ*g/kg), by 83.71 ± 10.86% (*P* < 0.001), 94.78 ± 6.65% (*P* < 0.001), and 96.02 ± 7.68% (*P* < 0.001) also attenuated met-AESP-induced hypotension by 50.62 ± 29.65% (*P* < 0.01), 41.46 ± 28.47% (*P* < 0.01) and 42.68 ± 28.22% (*P* < 0.01), respectively (see Figures 8(a), 8(b), and 8(c)).

### 3.7. Effects of eNOS Enzyme Blockage on Changes in MAP, SBP, DBP, and HR of Anaesthetized WKY

**Figure 9:**
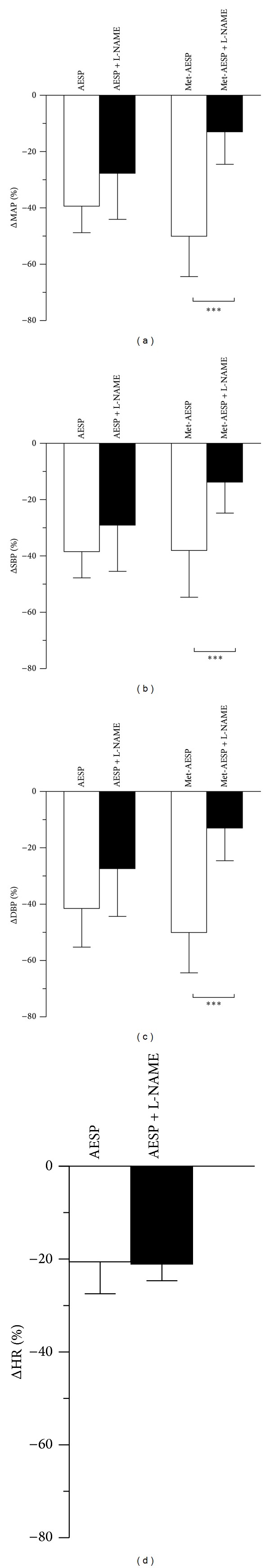
Effects of endothelial nitric oxide synthase enzyme blockage on changes in (a) MAP, (b) SBP, (c) DBP, and (d) HR by various treatments in WKY rats (*n* = 5). L-NAME: N-*ω*-nitro-l arginine methyl ester, ΔMAP/SBP/DBP/HR: changes of MAP/SBP/DBP/HR from baseline (expressed as mean percentage ± SD), AESP: aqueous extract of *S. polyanthum* leaves, met-AESP: residual methanolic extract of *S. polyanthum *leaves, WKY: Wistar-Kyoto rats, SHR: spontaneously hypertensive rats. ***P < 0.001.

Blockage of endothelial nitric oxide synthase, eNOS, with 20 mg/kg L-NAME caused significant attenuation in met-AESP-induced reductions of MAP, SBP, and DBP by 75.38 ± 19.32% (*P* < 0.001) 69.63 ± 19.79% (*P* < 0.001) and 75.38 ± 19.32% (*P* < 0.001) but not in AESP-induced reductions of MAP, SBP, DBP, and HR (see Figures 9(a), 9(b), 9(c), and 9(d)).

## 4. Discussion

This study has shown that both intravenous injections of AESP and met-AESP boluses induced significant dose-dependent hypotension while only the highest dose of AESP caused significant bradycardia. The occurrence of bradycardia, found to be accompanying the hypotensive effect of AESP, was common in few other ethnomedicinal plants such as *Tacca integrifolia* (Ker-Gawl). [26],* Musanga cecropioides* [27], and *Sida cordifolia *[25]. Nevertheless, there are some other ethnomedicinal plants such as *Andrographis paniculata* [23, 28] and* Bacopa monnieri* [29], elicited hypotensive effects without significant bradycardia as observed during met-AESP-induced hypotension. Besides the appearance of bradycardia for AESP, AESP and met-AESP differed in terms of onset time and sustainability of its action. The onset time for AESP to achieve maximal hypotension was faster than that for met-AESP in both WKY and SHR. AESP-induced hypotension was less sustained than met-AESP-induced hypotension in SHR. This may indicate different composition of active constituent(s) that perhaps acted on different pathways in mediating the hypotensive effects by both extracts. This study also showed that AESP-induced hypotension was more potent in WKY whereby it exhibited a comparable potency in SHR as compared to met-AESP-induced hypotension.

This study has shown that both extracts reduced MAP, SBP, and DBP of both WKY and SHR. However, the recovery time for AESP-induced hypotension and bradycardia was shorter in SHR as compared to in WKY. This can be explained by the preexistence of sympathetic overactivity in SHR as the over activity of sympathetic system in turn might cause a greater reflex pressor response to return the MAP, SBP, DBP, and HR levels to the baseline in SHR. This phenomenon is possible due to an altered number of adrenergic receptors or change in its responsiveness in SHR [30].

Further pharmacological antagonistic studies were then performed to elucidate the postulation that both extracts acted via different mechanism(s) of actions. Since the receptors of ANS are involved in sympathetic and parasympathetic controls of blood pressure and heart rate, their effects were further examined.

In this study, autonomic ganglion blockage was achieved by addition of hexamethonium, a nicotinic receptors blocking agent which blocks ion channels of the autonomic ganglia resulting in a blockage of the outputs of the sympathetic and parasympathetic pathways [31]. Since autonomic ganglion blockage had attenuated partially the AESP-induced hypotension, it might indicate a partial involvement of ANS in regulating its actions. Further blockages of the two peripheral receptors in ANS (cholinergic and *β*-adrenergic receptors) with their respective antagonists, propranolol and atropine, did not significantly affect neither AESP-induced hypotension nor its bradycardic effect. However, blockage of *α*-adrenergic receptors with phentolamine, a competitive blocker of *α*_1_- and *α*_2_-adrenergic receptors, had partially attenuated AESP-induced hypotension and bradycardia. Hence, it is suggested that AESP-induced hypotension and bradycardia in this study were partially acted via ANS, mediated by *α*-adrenergic receptors' pathways. It is known that *α*_1_-adrenergic receptor mediates vasoconstriction. On the other hand, *α*_2_-receptors play a prominent role in lowering blood pressure by inhibiting the synaptic release of neurotransmitter that mediates the renin production [32]. Alpha 2-adrenergic receptors stimulation also modulates vagally-induced baroreflex bradycardia [33]. Besides, hypotension with accompanying bradycardia is a common effect of *α*_2_-agonists such as clonidine, guanfacine, and *α*-methyldopa [33, 34]. Thus, it is plausible to suggest that AESP-induced hypotension was partially mediated by the *α*_2_-receptors.

As for met-AESP, blockage of autonomic ganglion significantly attenuated its hypotensive effect which may suggest that its action was primarily mediated *via* ANS. Blockages of the peripheral *β*-adrenergic receptors with propranolol, a nonselective *β*-adrenergic receptors' blocker [35], caused a significant attenuation in met-AESP-induced hypotension. Hence, it is suggested that met-AESP partially acted via *β*-adrenergic receptors of the ANS. Among the *β*-adrenergic receptors' subtypes, *β*_1_ subtype is involved in increasing the blood pressure and heart rate while *β*_3_ subtype was involved in lipolysis and not related to the regulation of blood pressure [36]. Thus, it is suggested that *β*_2_ subtype receptor which is responsible for smooth vessel relaxation [37] was involved in mediating hypotensive effect of met-AESP. Blockages of the peripheral muscarinic acetylcholine receptors with atropine, a competitive muscarinic acetylcholine receptors' blocker [38], also caused significant attenuations in met-AESP-induced hypotension. Hence, it is also suggested that, besides *β*-adrenergic receptors, met-AESP-induced hypotension was also partially mediated via muscarinic acetylcholine receptors. Moreover, the degree of attenuation by atropine was much greater than that by propranolol. Hence, it is suggested that met-AESP-induced hypotension was predominant on muscarinic acetylcholine receptor. Muscarinic acetylcholine receptor subtypes include M_1_, M_2_, M_3_, M_4_, and M_5_. M_1_ subtype is present in gastric parietal cells whereas M_4_ and M_5_ subtypes are not being described in blood vessels. M_2_ subtype may cause reduction of the cardiac output, blood pressure, and HR as a result of the decrement of firing rate at the sinoatrial node in the heart [39]. Thus, this subtype is unlikely to be involved since met-AESP did not cause any significant reduction of the HR. Therefore, it is suggested that the M_3_ subtype was possibly involved since its activation causes endothelium-dependent vasodilatation [40]. This study also showed that the action of met-AESP on ANS was less specific as compared to AESP.

Beta-2-adrenergic and M_3_-muscarinic acetylcholine receptors that are suggested to mediate met-AESP-induced hypotension are associated with endothelium-dependent vasodilatation or relaxation via increased synthesis of nitric oxide [37, 40, 41]. Therefore, another antagonistic study using L-NAME, a blocker of endothelial nitric oxide synthase (eNOS), was performed. It is found that met-AESP-induced hypotension was attenuated after blockage of eNOS while AESP-induced hypotension and bradycardia were not significantly attenuated after blockage of eNOS. These findings may support the suggestion of the partial involvement of *β*_2_-adrenergic and M_3_-muscarinic acetylcholine receptors in mediating met-AESP-induced hypotension. Besides, these findings may also suggest that AESP-induced hypotension did not involve direct nitric oxide generation.

Taken together, this study may serve as a preliminary verification for the traditional claim of using *S. polyanthum* leaves as a treatment of hypertension. The findings demonstrated that AESP-induced hypotension was more potent than met-AESP in WKY but both were comparably potent in SHR. AESP acted faster in both WKY and SHR. AESP exhibited comparable sustenance as met-AESP in WKY but it was less sustained in SHR. ANS partially mediates AESP actions via *α*-receptors and met-AESP via *β*-adrenergic and muscarinic acetylcholine receptors through NO generation. However, further works using much more subtype-specific antagonists should be done for much more comprehensive verification. Further studies are also warranted to investigate the active compound(s) in AESP and met-AESP responsible for their hypotensive effects.

## Figures and Tables

**Table 1:** ED_50_ values of AESP and met-AESP-induced hypotension.

Model of rats	ED_50_ values for the induced hypotension					
	AESP			Met-AESP		
	MAP	SBP	DBP	MAP	SBP	DBP
WKY	25.28 ± 2.33	21.80 ± 2.34	26.67 ± 2.34	54.58 ± 2.41	63.83 ± 2.37	72.14 ± 2.43
SHR	32.28 ± 2.53	26.32 ± 2.52	32.97 ± 2.58	33.05 ± 2.34	32.08 ± 2.36	32.25 ± 2.34

ED_50_ value: mean of dose ± SD that causes 50% maximal reduction of MAP, SBP, and DBP, calculated by GraphPad PRISM version 5.01 software by using nonlinear regression equation, MAP: mean arterial blood pressure, SBP: systolic blood pressure, DBP: diastolic blood pressure, AESP: aqueous extract of *S. polyanthum* leaves, met-AESP: residual methanolic extract of *S. polyanthum *leaves, WKY: Wistar-Kyoto rats, SHR: spontaneously hypertensive rats.

**Table 2:** Recovery time (in minutes) for 20, 30, 40, 70, and 100 mg/kg AESP and met-AESP in WKY and SHR.

		Recovery time (min)							
Dose (mg/kg)		AESP				Met-AESP			
		MAP	SBP	DBP	HR	MAP	SBP	DBP	HR
20	WKY	2	2	2	n.a	n.a	n.a	n.a	n.a
	SHR	1	1	2	n.a	n.a	n.a	n.a	n.a
30	WKY	3	3	3	n.a	n.a	n.a	n.a	n.a
	SHR	1	1	2	n.a	3	3	4	n.a
40	WKY	3	3	3	n.a	4	4	4	n.a
	SHR	2	2	2	n.a	4	4	4	n.a
70	WKY	4	4	4	n.a	4	4	4	n.a
	SHR	2	2	2	n.a	4	5	6	n.a
100	WKY	5	6	6	17	5	6	5	n.a
	SHR	3	4	4	5	4	4	5	n.a

Recovery time: the time when the averaged readings of MAP, SBP, DBP, and HR were no longer significantly different as compared to the initial baseline values. MAP: recorded mean arterial blood pressure in millimetres of mercury, HR: recorded heart rate in beats per minute, AESP: aqueous extract of *S. polyanthum* leaves, met-AESP: residual methanolic extract of *S. polyanthum *leaves, WKY: Wistar-Kyoto rats, SHR: spontaneously hypertensive rats, n.a: there were no changes observed in the baselines of MAP, SBP, DBP, and HR upon administration of the respective doses.
